# Thrombus inside the channel of patent foramen ovale revealed by optical coherence tomography imaging in a patient with myocardial infarction

**DOI:** 10.1093/ehjcr/ytae304

**Published:** 2024-07-08

**Authors:** Xing-ye Wang, Lu He, Xue-gang Xie, Xiao-qin Liu, Yu-shun Zhang

**Affiliations:** Department of Structural Heart Disease, The First Affiliated Hospital of Xi’an Jiaotong University, No. 277 Yanta West Road, Xi’an 710068, China; Department of Structural Heart Disease, The First Affiliated Hospital of Xi’an Jiaotong University, No. 277 Yanta West Road, Xi’an 710068, China; Department of Structural Heart Disease, The First Affiliated Hospital of Xi’an Jiaotong University, No. 277 Yanta West Road, Xi’an 710068, China; Department of Structural Heart Disease, The First Affiliated Hospital of Xi’an Jiaotong University, No. 277 Yanta West Road, Xi’an 710068, China; Department of Structural Heart Disease, The First Affiliated Hospital of Xi’an Jiaotong University, No. 277 Yanta West Road, Xi’an 710068, China

**Keywords:** Myocardial infarction, Patent foramen ovale, *In situ* thrombus, Case report

## Abstract

**Background:**

Myocardial infarction (MI) caused by patent foramen ovale (PFO)-based paradoxical embolism is rare, and there are few case reports in the literature.

**Case summary:**

Here, we report a case of MI in which optical coherence tomography revealed *in situ* thrombi in the PFO channel.

**Discussion:**

In addition to paradoxical embolism, *in situ* thrombus may also be one of the pathogenic mechanisms of PFO in patients with MI.

**Learning points**

- In young patients with myocardial infarction, *in situ* thrombus attached to patent foramen ovale may be the source of coronary artery embolism.
- Optical coherence tomography can clearly visualize microthrombus and can be used to find the source of thrombus.

## Introduction

Patent foramen ovale (PFO) occurs in 20–34% of the general population, with most of them being asymptomatic.^1^ The closure of PFO is maintained through the pressure gradient between the left and right atria. However, during actions such as coughing, squatting, or defecating, this pressure gradient can reverse owing to a transient increase in right atrial pressure, leading to opening of PFO and occurrence of a right-to-left shunt (RLS). Consequently, substances such as microemboli from the venous system can enter the arterial system,^2^ a phenomenon called paradoxical embolism. Patent foramen ovale is most commonly associated with paradoxical embolism and clinically plays an essential role in the pathogenesis of several conditions, including stroke, migraine, and decompression sickness.^1^

In addition to paradoxical embolism, Yan *et al*.^3,4^ found thrombi in the PFO fissure using optical coherence tomography (OCT) in patients with cryptogenic stroke but not in asymptomatic PFO patients. The authors speculated the involvement of *in situ* thrombi in the pathogenesis of stroke. Patent foramen ovale can lead to intracranial infarction and rare extracranial embolisms such as myocardial infarction (MI), which has a rare incidence of ∼0.65%.^5^ However, *in situ* thrombi inside the PFO channel have rarely been reported in patients with MI.^6^ Here, we present a case of MI with thrombi inside the PFO channel detected using OCT.

## Summary figure

**Figure:**
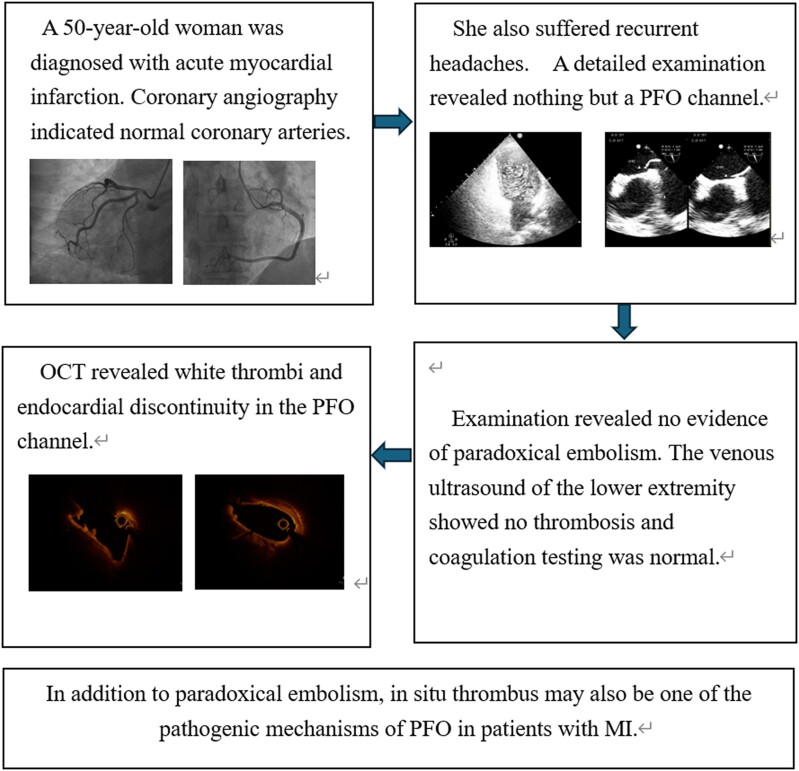


## Case presentation

A 50-year-old woman who presented with one episode of chest pain lasting 3 min, accompanied by a 2-year history of hypertension, was admitted to the outpatient clinic of a local hospital, where electrocardiogram revealed normal findings, and she discharged home without medication.

One week later, she returned to the local hospital’s emergency department for sudden and severe chest pain persisting without relief for 40 min. Electrocardiogram indicated ST-segment elevation in the V1 to V4 leads and elevated myocardial enzyme spectrum. She was diagnosed with acute MI, and emergency coronary angiography was conducted, but no apparent stenosis was found in the vessels. Unfortunately, no other abnormalities were found at the local hospital. She was discharged with routine medication for primary prevention of coronary heart disease, including aspirin, clopidogrel, and atorvastatin.

**Figure 1:**
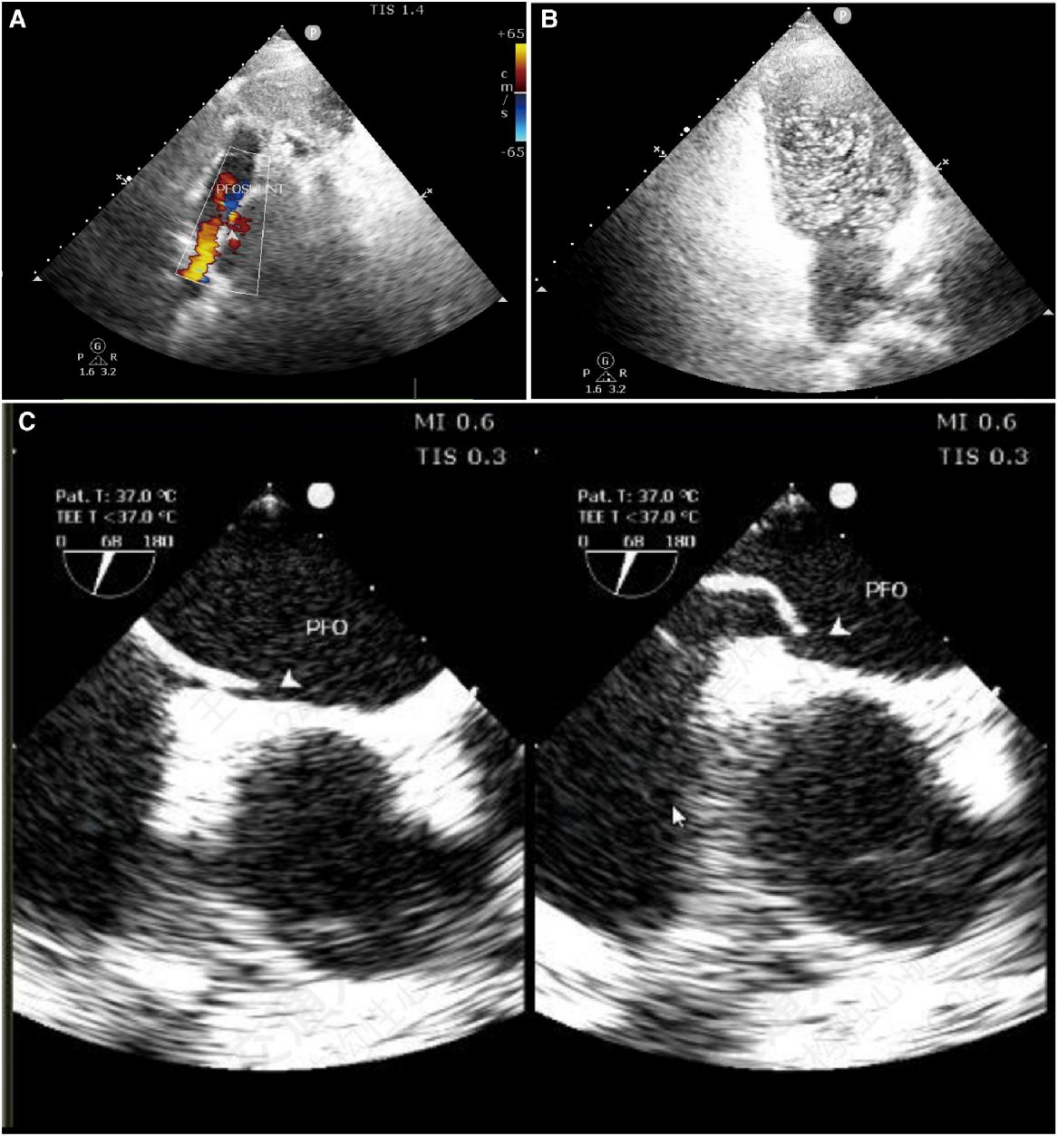
Images of echocardiogram: (*A*) Oblique shunt on atrial septum was revealed by transthoracic echocardiography; (*B*) contrast transthoracic echocardiography shows a large right-to-left shunt; (*C*) anatomy of patent foramen ovale indicated by transoesophageal echocardiography.

**Figure 2:**
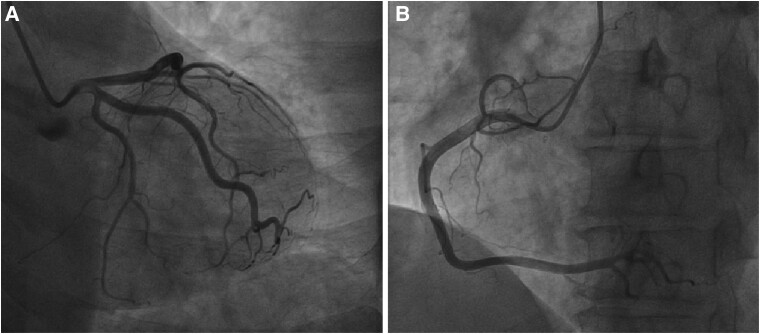
*A* and *B* present normal coronary angiography images.

Two months later, she experienced headache attacks approximately once or twice a week, prompting admission to our hospital. A detailed medical history revealed that her headaches had been occurring for 2 years, combined with well-controlled high blood pressure. The coexistence of MI with normal coronary arteries and recurrent headaches led us to explore a potential association, prompting a series of diagnostic tests. Cranial magnetic resonance imaging showed no abnormalities (see Supplementary material online, Figure). After 24 h Holter monitoring, no atrial fibrillations or other arrhythmias were revealed. Transthoracic echocardiography revealed no abnormalities except for oblique shunt over the atrial septum (*Figure 1A*). Contrast transthoracic echocardiography examination indicated sustained RLS (*Figure 1B*). Transoesophageal echocardiography showed a PFO channel with 2.6 mm height and 9.3 mm length (*Figure 1C*). Venous ultrasound of the lower extremity showed no thrombosis, and coagulation testing yielded normal findings. The Caprin Assessment was used to evaluate the thromboembolic risk, and the patient received a score of 1 (for age 50 years), placing her in the low-risk group. Based on the patient’s history, a possible coronary artery embolism secondary to PFO was considered. Before initiating PFO occlusion treatment, coronary angiography was repeated at our centre, confirming normal coronary artery (*Figure 2A* and *B*).

**Figure 3:**
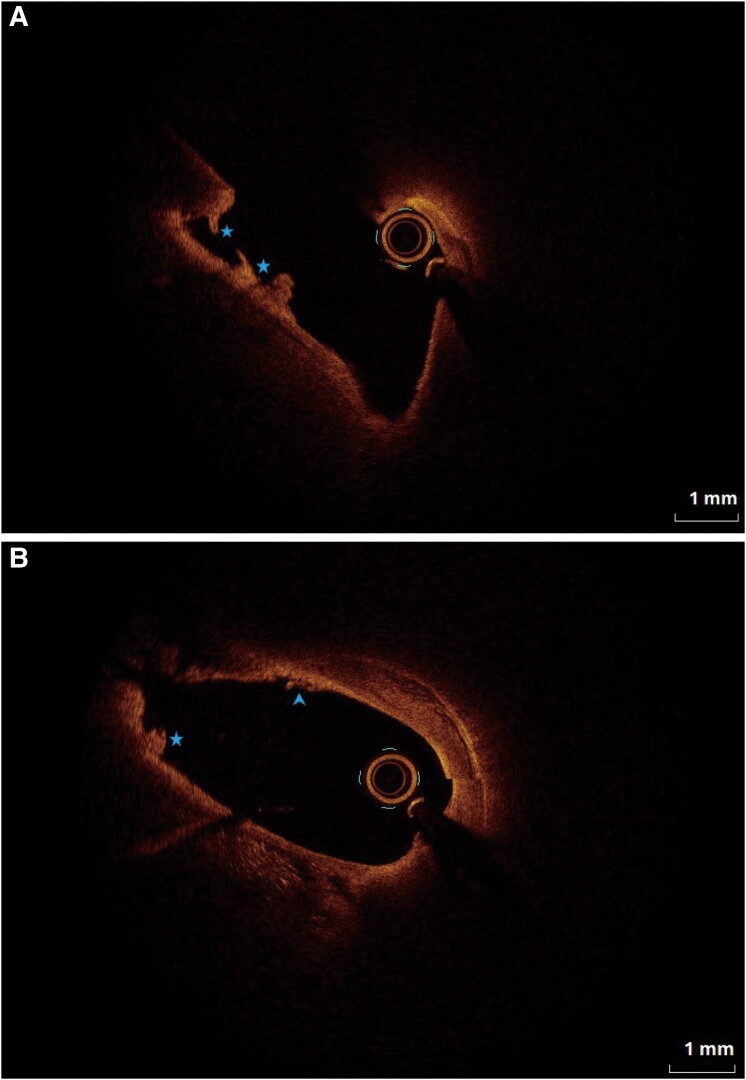
(*A*) and (*B*) present images of optical coherence tomography, star refers to white thrombi and arrow endocardial discontinuity.

Given the absence of evidence supporting paradoxical embolism, we suspected *in situ* thrombosis. Optical coherence tomography was performed with the patient’s consent. The OCT catheter was advanced ∼1 cm beyond the left atrial opening of the foramen ovale fissure. When the contrast agent was injected through a guiding catheter to clear the blood in the fissure, the catheter was withdrawn to the right atrial of the PFO tunnel (see Supplementary material online, *Video S1*). Optical coherence tomography revealed three white thrombi in the channel, with a calculated thrombus area of 0.19 mm^2^ and total thrombus volume of 0.13 mm^3^. Additionally, we found an endocardial discontinuity in the tunnel (*Figure 3A* and *B*, Supplementary material online, *Video S2*).

To prevent recurrence of embolic events, we chose a 25 mm Amplatzer PFO closure device to close her PFO. At the 6-month follow-up, the patient was in good condition, with resolution of headache and no recurrence of chest pain. The patient provided consent for the publication of this report and its associated data.

## Discussion

MI is a serious cardiovascular emergency and the leading cause of death worldwide.^7^ Reduced blood flow due to rupture or erosion of vulnerable plaques and formation of thrombus in the coronary artery are the main mechanisms of its pathogenesis.^8,9^ Although MI caused by a paradoxical embolism is a rare, exclusive diagnosis, other potential causes of MI, such as coronary atherosclerosis, vasospasm, heart valve defects, and atrial fibrillation, should be systematically excluded. When paradoxical embolism is highly suspected after eliminating the above reasons, it is necessary to find evidence of an abnormal embolism, including an abnormal intracardiac or extracardiac shunt. MI caused by an intracardiac shunt mainly involves an atrial septal defect and PFO.^2^ Unlike other intracardiac shunts, PFO exhibits slow or stable blood flow in the unhealed fissure between the septum primum and septum secundum, creating a conducive environment for *in situ* thrombus formation. These thrombi may subsequently enter the arterial circulation through an RLS, causing embolism events.

In patients with PFO-related stroke, the incidence of detectable thrombus in the lower limb and pelvic veins is only ∼11%,^10^ indicating that the source of most clots remains unknown. An *in situ* thrombus in the PFO tunnel has always been a hypothetical source of thrombus.^11^ Using OCT, Yan *et al*. found that, in addition to being a thrombus pathway itself, the tunnel of PFO-related stroke patients is the site for *in situ* thrombus formation. In contrast, no thrombi were found in the tunnel of asymptomatic PFO patients, and *in situ* thrombi are likely to be the pathogenic mechanism of PFO.^4^ As a pioneering study, the researchers only included patients with PFO-related stroke and migraine, excluding those with extracranial infarction caused by PFO, which is rare in clinical setting.^5^*In situ* thrombi inside the PFO channel in patients with MI have rarely been reported.^6^ In our patient, white thrombi were attached to the PFO channel, and the endocardial surface was irregular, which is consistent with the conclusion of Yan *et al.*^4^

Based on the primary mechanism of MI pathogenesis, clinical treatments for acute MI mainly focus on restoring blood flow, including primary percutaneous coronary intervention and thrombolysis.^12^ However, if MI is due to a paradoxical embolism, the management strategy may not be completely consistent. Manual aspiration thrombectomy and avoidance of stent implantation is beneficial. Additionally, beta-blockers and angiotensin-converting enzyme inhibitors may be used in cases of reduced left ventricular ejection fraction, whereas antiplatelet or statin therapy may not be necessary. Anticoagulant therapy has been used to treat venous thrombosis.^13^ Despite the absence of evidence from randomized controlled trials on PFO closure in MI induced by PFO, finding from a large multicentre case–control study have shown equivalent efficacy of PFO closure in treating peripheral embolisms, including MI, compared to intracranial infarction.^14^ Blocking the RLS caused by the PFO may emerge as the preferred strategy, supported by remission of symptoms in patients after PFO closure.

## Conclusion

In patients with MI considered to be caused by PFO, *in situ* thrombus within the PFO fissure can be revealed on OCT, which represents another pathogenic mechanism that deserves further investigation.

## Supplementary Material

ytae304_Supplementary_Data

## Data Availability

All available data was presented within the manuscript.

## Lead author biography

Professor, MD, Vice President of Cardiovascular Hospital, and leader of the Department of Structural Heart Disease, The First Affiliated Hospital of Xi’an Jiaotong University.

## Supplementary material

**Consent:** Written informed consent to publish the clinical details and images of the patient was obtained, in line with the COPE best practice guidelines.

**Funding:** None.
