# Supplementary figures and images for: Thrombus inside the channel of patent foramen ovale revealed by optical coherence tomography imaging in a patient with myocardial infarction
Source: Eur Heart J Case Rep. 2024 Jul 8;8(7):ytae304. doi: 10.1093/ehjcr/ytae304 (PMC11287203; doi:10.1093/ehjcr/ytae304)

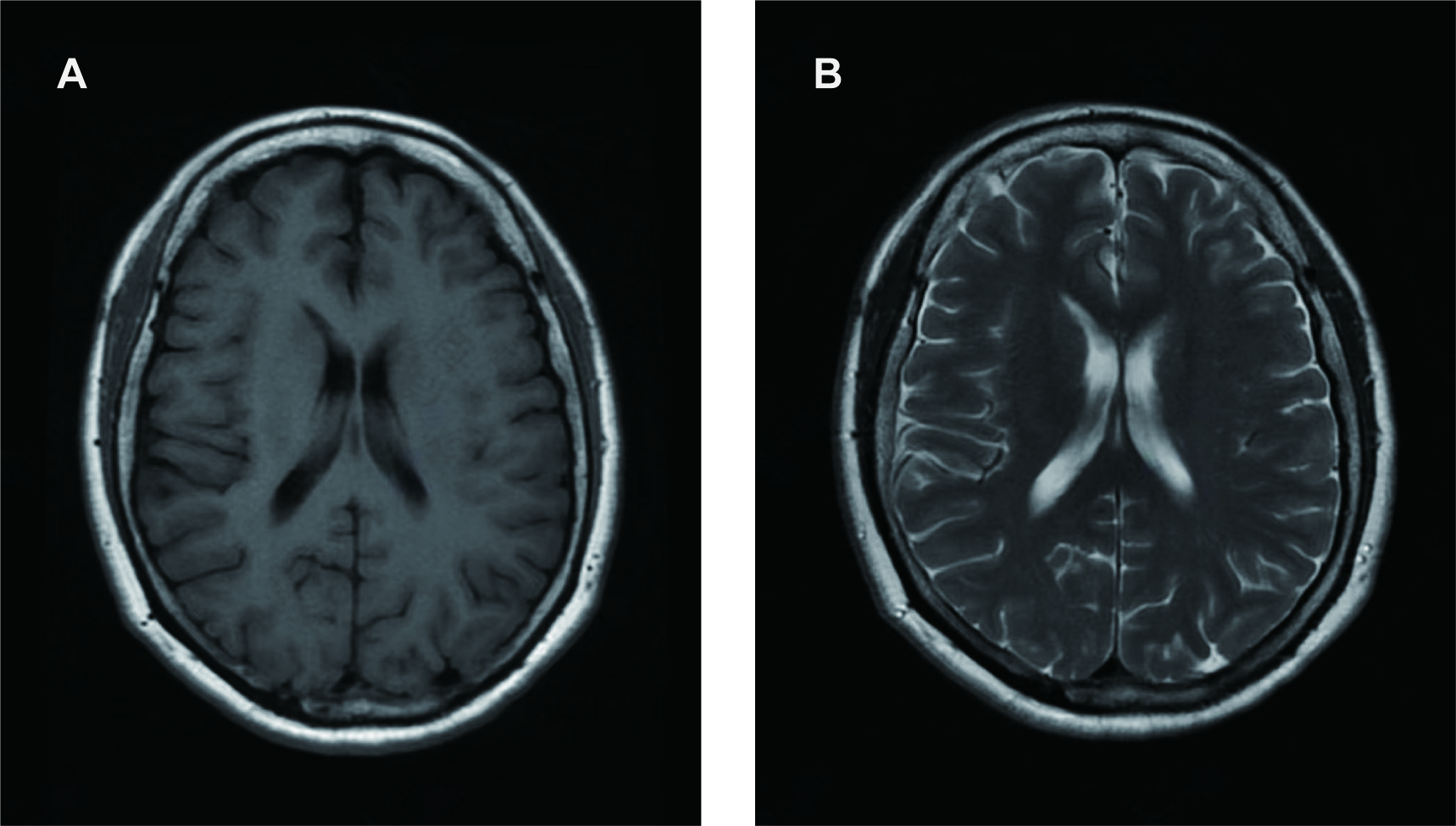

Supplement: ytae304_Supplementary_Data [file ytae304_supplementary_data.zip › Supplementary Figure.tif]
